# Serum VEGF: Diagnostic Value of Acute Coronary Syndrome from Stable Angina Pectoris and Prognostic Value of Coronary Artery Disease

**DOI:** 10.1155/2020/6786302

**Published:** 2020-01-10

**Authors:** Anan Huang, Xin Qi, Yameng Cui, Yulin Wu, Shiqi Zhou, Mingyin Zhang

**Affiliations:** ^1^Nankai University School of Medicine, Tianjin 300071, China; ^2^Department of Cardiology, Tianjin Union Medical Center, Nankai University Affiliated Hospital, Tianjin 300121, China; ^3^Graduate of School, Tianjin University of TCM, Tianjin 300193, China

## Abstract

**Background:**

Although the level of serum vascular endothelial growth factor (VEGF) is elevated in coronary artery disease (CAD) patients, its potential role in acute coronary syndrome (ACS) or stable angina pectoris (SAP) patients remains unclear.

**Objectives:**

To evaluate diagnostic accuracy of serum VEGF in determining ACS patients from SAP and analyze the association of serum VEGF with coronary artery lesions in SAP or the GRACE score in ACS, which is involved in the poor prognosis of low serum VEGF.

**Methods:**

248 CAD patients and 48 healthy subjects were enrolled in this study. Serum VEGF levels were detected by using ELISA. The Gensini score or GRACE score was calculated among SAP or ACS patients. All the patients were followed up for a period of 12 months (mean: 10.77 months).

**Results:**

VEGF serum concentrations were higher in the ACS subgroup than in the SAP subgroup (*P* < 0.001) with diagnostic accuracy of ACS from SAP (AUC: 0.667, sensitivity: 68.5%, specificity: 60.1%, *P* < 0.001). Patients with high risk of Gensini score showed reduced VEGF levels (*P* < 0.001) accompanied by a negative correlation (*r* = −0.396, *P* < 0.001). Patients with a higher GRACE score indicated lower VEGF levels (*P* < 0.001). Low serum VEGF was one of the potential risk factors with adjusted HR of 0.531 (*P*=0.048).

**Conclusion:**

Serum VEGF exhibits efficient diagnostic value for detection of ACS from SAP with a cutoff value of 648.75 pg/mL. Low serum VEGF indicates severe coronary artery lesions and a higher GRACE score, which suggests poor clinical outcomes.

## 1. Introduction

Coronary artery disease (CAD), emerged mainly as cardiovascular disease, caused almost 8 × 10^6^ deaths per year with an increase of 47.5% from 1990 to 2013 around the world [1]. The estimated costs for the treatment of CAD and myocardial infarction (MI) were 12.1 and 9 billion, respectively, in 2013 and will increase by as much as 100% in next 20 years [2]. Although multiple drug and surgical treatments have been performed in clinical practice, CAD still presents poor prognosis compared with other kinds of malignant diseases [3, 4]. As one of the initial factors, imbalance in coronary blood flow between supply and demand induces myocardial ischemia. The compensatory capacity of angiogenesis in ischemic myocardium might correlate with severity of coronary atherosclerosis and even clinical prognosis.

Recently, interests keep growing on utilizing several serum biomarkers to estimate severity of coronary artery lesions. Vascular endothelial growth factor (VEGF), emerged as the most important cytokine in inducing angiogenesis, stimulates the formation of neovessels and promotes the foundation of blood flow. VEGF expression could attenuate necrosis of cardiomyocytes and heart remodeling [5]. Previous literature demonstrated that decreased VEGF levels could predict the occurrence of CAD including stable angina pectoris (SAP) and acute coronary syndrome (ACS) [6, 7]. VEGF was mainly synthesized by ischemic cardiomyocytes in SAP. On the contrary, cardiac inflammatory response played an essential role in upregulating VEGF expression in ACS. Few data estimated the essential role in ACS and SAP patients. The correlation between serum VEGF and severity of coronary artery lesions remains unknown, and in addition, serum VEGF levels under distinct stratifications of GRACE score in ACS were also unclear.

In the present study, we hypothesized differentiated expression of serum VEGF in ACS and SAP patients. Serum VEGF levels might became negative in association with the severity of coronary artery lesions in SAP, and low VEGF might indicate higher GRACE score. Low VEGF could be one of the essential risk factors for poor clinical outcomes in CAD patients.

## 2. Methods

### 2.1. Study Population

248 CAD patients with typical discomforts in the precordial area including chest pain or chest distress were admitted to the department of cardiology in the Tianjin Union Medical Center (Tianjin, PR. China) from September 2016 to September 2018. 48 serum samples from healthy controls without CAD, hypertension, and diabetes mellitus (DM) were collected from the physical examination center in the Tianjin Union Medical Center. All patients required invasive evaluation and underwent coronary artery angiography (CAG) using the standard technique. The diagnosis of CAD was based on clinical characteristics, symptoms, and the results of CAG. In brief, CAD was characterized as the vicinity of luminal width narrowing of 50% in the left anterior descending artery (LAD), left circumflex (LCX), or right coronary artery (RCA). The diagnostic criteria of CAD were in accordance with the relevant guidelines by two experienced cardiologists [8]. CAD patients were further divided into SAP subgroup or ACS subgroup based on the guidelines [8–10]. Exclusion criteria for the present study were stroke, cardiomyopathy, severe trauma, preexisted MI, stent implantation, coronary artery bypass graft (CABG), severe cardiac, renal or liver dysfunction, autoimmune diseases, malignant diseases, and other inflammatory states. The Ethical Committee board of the Tianjin Union Medical Center approved this study protocol.

### 2.2. Clinical Data Collection

Clinical data collection was recorded by one of the specific cardiologists and then rechecked by the other one. The patients' demographic characteristics and preexisting comorbidities were collected at the time of hospitalization. In-hospital biochemical data including hemoglobin A1c (HbA1c), creatine kinase-MB (CK-MB), troponin I (TnI), total cholesterol (TC), triglyceride (TG), high-density lipoprotein-cholesterol (HDL-c), low-density lipoprotein-cholesterol (LDL-c), and creatinine (Cr) were detected by using the biochemistry analyzer (Abbott Architect c16000 System, Chicago, U.S). Briefly, HbA1c levels were determined by using a high-pressure liquid chromatography instrument (Roche Diagnostics, Mannheim, Germany), and TC levels were determined by the CHOD-PAP method (cholesterol reagent; Shanghai Fosun Long March Medical Science Co., Ltd). The GPO-PAP method was used to test TG levels (triglycerides reagent; Shanghai Fosun Long March Medical Science Co., Ltd). In addition, the HDL-c (HDL- cholesterol reagent kit; Shanghai Fosun Long March Medical Science Co., Ltd) and LDL-c (LDL-cholesterol reagent kit, Shanghai Fosun Long March Medical Science Co., Ltd) levels were determined by the clearance method, respectively. In addition, TnI levels were determined by chemiluminescence microparticle immunoassay (CMIA) using ARCHITECT TnI kits (Abbott, Chicago, U.S). Cr levels were tested by ammonia iminohydrolase-PAP (Cr reagent kit, Shanghai Fosun Long March Medical Science Co., Ltd). All of these biochemical data were collected in the Core Laboratory of Tianjin Union Medical Center within first 24 hours after hospitalization. Left ventricular ejection fraction (LVEF) was calculated by a diagnostic medical sonographer. Hypertension was recognized as blood pressure over 140/90 mmHg in two different time points or taking any antihypertensive treatment earlier. DM was defined as fasting blood glucose over 126 mg/dl or taking any glucose-lowering treatment earlier.

### 2.3. Measurement of Biomarkers

Blood samples were collected from the median cubital vein and centrifuged at 3600 rpm for over 10 minutes to obtain the serum, and it was stored at −80°C for further analysis. All blood samples were tested by only one freeze-thaw cycle. Serum VEGF levels were determined by specific human vascular endothelial growth factor (VEGF) enzyme-linked immunosorbent assay (ELISA) (no. DRE19920, QIYI Biological technology. Co., LTD. Shanghai, China) kits with a coefficient variation (CV) of 9%.

### 2.4. Gensini Score

The Gensini score was calculated for all SAP patients. Calculation of the Gensini score has been reported in the literature [11]. In brief, extension of luminal narrowing and its geographic importance were taken into account to assess the severity of coronary artery lesions. Risk stratification of the Gensini score was classified into 3 categories including low-risk, medium-risk, and high-risk subgroups by the tertiles of the Gensini score in SAP patients [12–14].

### 2.5. GRACE Score

For those ACS individuals, the GRACE score is an in-hospital or postdischarge cardiovascular risk evaluation model [15, 16]. The risk evaluation model included variables of age, heart rate, serum creatinine, Killip's class, cardiac arrest at admission, elevated cardiac markers, and ST-segment deviation. The GRACE score was further systematically classified into 3 categories of low risk (∼108), medium risk (109–140), and high risk (140∼).

### 2.6. Follow-Up Evaluation and Study End Points

All the patients were followed up for a period of 12 months (mean: 10.77 months) after discharge by clinic visits, telephone interviews, and analysis of readmission. The primary end points included all-cause mortality and cardiovascular mortality. The secondary end points included the occurrence of ischemic events, reoccurred chest pain, PCI, or CABG. Adverse events were recorded including primary and secondary end points. The follow-up was terminated after initially reaching any end points.

### 2.7. Statistics

Data were analyzed using the statistical package for social sciences (SPSS Inc., Chicago, IL) version 17.0. Discrete variables were expressed as numbers and percentages. The mean ± SD or median with an interquartile (IQ) range between 25% and 75% (Q25–Q75) based on the normality for continuous variables. Normality for the continuous variables was performed by the Kolmogorov–Smirnov (K-S) tests. Categorical variables were analyzed by the chi-square tests. To compare groups, we used the Mann–Whitney *U*-test or Kruskal–Wallis test for nonnormally distributed continuous variables. The receiver operating characteristics (ROC) curve was depicted for detection of ACS from SAP. The optimal cutoff value was determined by the point on the curve with minimum distance from the left upper corner of the unit square. The hazard ratio (HR) and 95% confidence intervals (95% CI) were estimated by univariate and multivariate Cox regression models for predicting clinical prognosis. Potent variables with a *P* value of less than 0.1 on the univariate analyses were entered into a multivariate analysis. The unadjusted rate of cumulative cardiovascular adverse events was estimated using the Kaplan–Meier curve, and log-rank tests were analyzed across groups. In all analyses, statistical significance was accepted at *P* < 0.05.

## 3. Results

**Figure 1:**
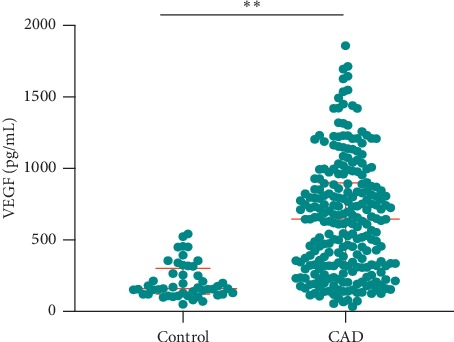
Serum VEGF levels in CAD patients and healthy controls. (a) VEGF serum concentrations in CAD patients were significantly higher than those in healthy controls (^*∗∗*^*P* < 0.01). Values are median (interquartile range). VEGF: vascular endothelial growth factor; CAD: coronary artery disease.

In this study, two hundred and forty eight subjects were recruited in the CAD group and forty eight subjects were enrolled as healthy controls. Baseline characteristics of this study population were demonstrated in Table 1. Of the 248 CAD patients, 45 (18.1%) experienced adverse events (10 patients died and 38 patients underwent reoccurred chest pain, PCI, or CABG) during a median of 10.77 months follow-up. The average age was 61.54 years and approximately 42.3% were male among the CAD patients, which presented no difference with healthy controls (*P* > 0.05). Several cardiovascular risks including hypertension, diabetes mellitus, and smoking were recorded in CAD patients, while none of these cardiovascular risks were found in all of the healthy controls. The biochemical data showed HbA1c, CK-MB, TnI, TG, and HDL-c levels in the CAD patients were higher than those in the healthy controls (*P* < 0.05), while no differences were demonstrated in Cr, TC, and LDL-c levels (*P* > 0.05). LVEF values in CAD patients were comparable with those in healthy controls (*P* > 0.05). In this study, appropriately 100 subjects (40.3%) underwent PCI therapy. VEGF serum median concentrations of CAD patients and healthy controls were 645.57 and 160.93 pg/mL (*P* < 0.001) (Figure 1).

**Figure 2:**
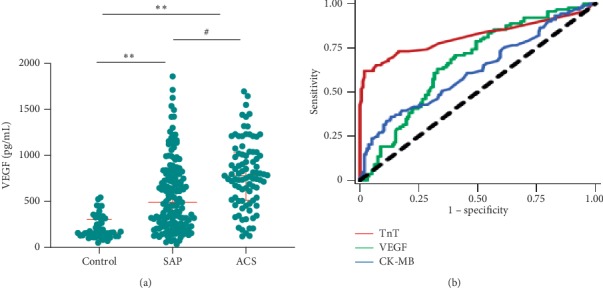
Diagnostic performance of serum VEGF levels for detection of ACS against SAP. (a) Comparison of serum VEGF levels in healthy controls, SAP, and ACS subgroups. Median VEGF levels in SAP and ACS subgroups increased with respect to healthy controls (^*∗∗*^*P* < 0.01). Median VEGF levels were elevated in the ACS subgroup compared to the SAP subgroup (^#^*P* < 0.01). (b) Receiver operating characteristics (ROC) curves for VEGF, TnI, and CK-MB for the detection of ACS. Data were expressed as median (interquartile range). SAP: stable angina pectoris; ACS: acute coronary syndrome; VEGF: vascular endothelial growth factor; TnI: troponin I; CK-MB: creatine kinase-MB.

To estimate the essential role of VEGF in discriminating ACS from SAP, all of the CAD patients were split into two subgroups of SAP and ACS. The median VEGF concentrations were significantly higher in the SAP subgroup 492.10 pg/mL (239.23, 824.81 pg/mL) than 779.28 pg/mL (513.22, 1028.16 pg/mL) in the ACS subgroup (Figure 2(a)). We further evaluate the diagnostic performance of VEGF levels for detection of ACS against SAP. The receiver operating characteristic (ROC) curve could help to determine ACS from SAP. We depicted ROC curves of VEGF and another two myocardial injury biomarkers of TnT and CK-MB (Figure 2(b), Table 2). Throughout the ROC curve analysis, the area under the ROC curve (AUC) of VEGF used to predict CAD was 0.667 (sensitivity: 68.5%; specificity: 60.1%), which was remarkably powerful compared to CK-MB with AUC of 0.622 (sensitivity: 60.7% and specificity: 54.4%).

**Figure 3:**
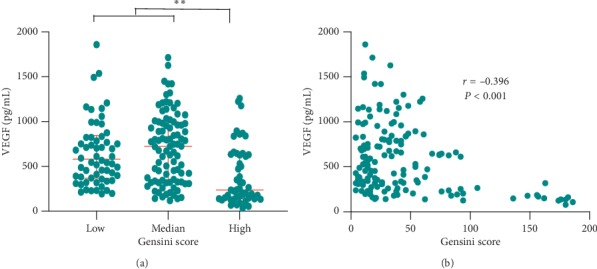
The association between VEGF levels and the Gensini score in SAP patients. (a). Comparison of VEGF serum concentrations under distinct risk stratifications of the Gensini score in the SAP subgroup. Patients with high Gensini score exhibited decreased VEGF levels, in contrast to patients with low and medium risk subgroups of Gensini score (^*∗*^*P* < 0.05). (b) Spearman correlation of VEGF levels and the Gensini score in the SAP subgroup. VEGF correlated negatively with the Gensini score in SAP patients (*r* = −0.396, ^*∗∗*^*P* < 0.001). Data are presented as median (interquartile range). SAP = stable angina pectoris; VEGF: vascular endothelial growth factor.

We calculated the Gensini score for each patient. We analyzed the potential value of VEGF in evaluating the severity of coronary artery lesions. Risk stratification of the Gensini score was built with low risk, medium risk, and high risk in line with the tertiles of 17 and 44 in SAP patients. Patients with high risk presented lower serum VEGF levels in contrast to higher serum VEGF levels of patients with low risk or medium risk (median: 237.38 vs 568.10 or 673.15 pg/mL, *P* < 0.001) (Figure 3(a)). We also performed the spearman rank correlation analysis in all SAP patients, and serum VEGF was negatively correlated with the Gensini score (*r* = −0.396, *P* < 0.001) (Figure 3(b)). These results suggested that low VEGF levels might imply a poor clinical prognosis in SAP patients.

**Figure 4:**
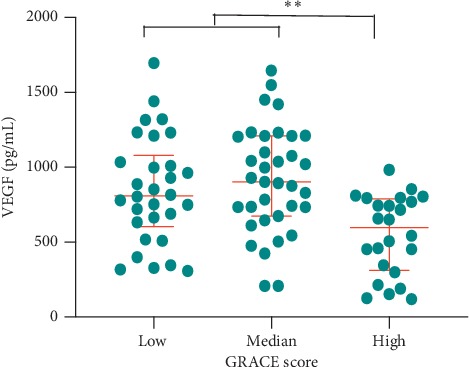
Comparison of VEGF levels under distinct risk stratifications of the GRACE score in ACS patients. Patients with high risk showed reduced VEGF levels compared to patients with low risk and medium risk (^*∗*^*P* < 0.05). Data are presented as median (interquartile range). VEGF: vascular endothelial growth factor; ACS: acute coronary syndrome.

The GRACE score was also calculated for each ACS patient to evaluate cardiovascular risk during hospitalization or after discharge. According to the risk stratification of the GRACE score, patients with high risk (GRACE score > 140) presented lower VEGF than another two subgroups of low risk (GRACE score ≤ 108) and medium risk (GRACE score between 109 and 140) (median: 596.83 pg/mL vs 809.99 pg/mL vs 903.05 pg/mL, *P* < 0.001) (Figure 4). This result indicated that low VEGF levels might be associated with high cardiovascular risk.

**Figure 5:**
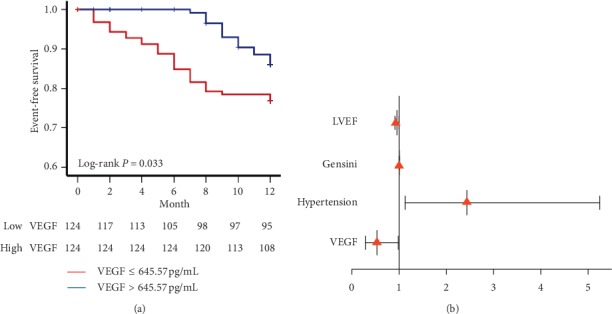
Prognostic value of serum VEGF in CAD patients. (a) Kaplan-Meier curves of distinct VEGF levels in predicting cardiovascular outcomes. VEGF levels below 645.57 pg/mL indicated a poor clinical prognosis. The *P* value indicates the difference between low VEGF levels and elevated VEGF levels. (b) VEGF and risk of cardiovascular adverse events. The forest plot of hazard ratios for cardiovascular adverse events in CAD patients. The *X*-axis indicated the hazard ratio and 95% confidence intervals. VEGF: vascular endothelial growth factor; CAD: coronary artery disease; LVEF: left ventricular ejection fraction.

Adverse events were recorded including primary and secondary end points. The follow-up was terminated after initially reaching an end point. All of the CAD patients were divided into two subgroups based on the median value of VEGF (645.57 pg/mL). During a mean follow-up period of 10.77 months, appropriately 29 patients (23.3%) with low VEGF and 16 patients (6.5%) with high VEGF had adverse events. In the Kaplan-Meier curves, we found that patients with lower serum VEGF levels showed a poor prognosis (*P*=0.033) (Figure 5(a)). To assess whether serum VEGF might become one of the potential prognostic factors, univariate and multivariate Cox analyses were performed (Table 3, Figure 5(b)) to determine associations between adverse events and studied variables. In univariate Cox analysis, we found that LVEF (RR: 0.954, 95% CI: 0.914–0.996; *P*=0.033), and hypertension (2.831, 95% CI: 1.318–6.080; *P*=0.008), Gensini score (1.006, 95% CI: 1.002–1.009; *P*=0.001), and low VEGF (0.522, 95% CI: 0.284–0.962; *P*=0.037) could be further brought into multivariate analysis. After adjusting for all these factors, low VEGF (RR: 0.531, 95% CI: 0.286–0.985; *P*=0.048) could be independent risk factors for predicting cardiovascular adverse events.

## 4. Discussion

The main results of this study are as follows: (1) serum VEGF increased in ACS patients compared to the SAP patients, which were both higher than healthy controls. (2) Low VEGF was negatively associated with the Gensini score. (3) VEGF serum concentrations decreased significantly in the high-risk subgroup of the GRACE score. (4) Low VEGF predicted a poor clinical prognosis. The novelty of this study is that we revealed serum VEGF differed between SAP and ACS which is conductive to distinguish ACS from SAP. In addition, our study found the negative correlation between serum VEGF and the Gensini score in SAP, and differential VEGF expression was found under distinct stratifications of the GRACE score in ACS. Patients with low VEGF showed increased occurrence rate of adverse events.

VEGF, emerged as one of the key regulators of angiogenesis, plays a crucial role in the whole pathophysiological process of wound healing, tumor growth, and myocardial ischemia [17]. Several observational studies have revealed that VEGF from peripheral blood decreased significantly in CAD patients, compared to healthy subjects [7, 17]. While few studies investigated the differential expression of serum VEGF between ACS and SAP [6, 18–20], in this study, serum VEGF levels in SAP or ACS were increased compared to healthy controls. Patients presented elevated serum VEGF levels in ACS patients by comparison to SAP patients. Moreover, the ROC curve suggested that serum VEGF could help to determine the detection of ACS from SAP, which could be used in clinical practice.

Myocardial tissue activated VEGF expression under stimuli of hypoxia in CAD with alternative mechanisms in SAP and ACS. Of ACS patients, unstable atherosclerotic plaque gives rise to inflammatory response which induced the expression of VEGF in cardiomyocytes and releases into peripheral blood. Alternatively, synthesis of VEGF is associated with severity of coronary atherosclerosis which may be in the light of endothelial dysfunction in patients with SAP [7]. Therefore, our study further investigated the essential role of serum VEGF in SAP and ACS.

The Gensini score was calculated through analyzing the results of angiographic data as one of the crucial models for assessing severity of coronary artery lesions [12]. This negative correlation between VEGF and the Gensini score draws our attention in SAP patients. Previously, the literature did not verify this association between serum VEGF and coronary artery lesions [20, 21]. Our data revealed that serum VEGF showed a negative correlation with Gensini score, and patients under high risk of Gensini score possessed lower VEGF levels. Patients with high Gensini score might indicate more severe coronary artery lesions or endothelial dysfunction, which could be related to insufficient VEGF. In our study, we analyzed this association in SAP to exclude inflammatory response from unstable atherosclerotic plaque of ACS. Besides the association between SAP and VEGF, the association between serum VEGF and GRACE score was also investigated. For those patients with ACS, risk stratification can assist cardiologists in making clinical decisions and predict prognosis during hospitalization or after discharge. Risk stratification of the GRACE score can predict cardiovascular risk within 6 months after discharge in ACS patients [15, 16, 22]. No literature has analyzed this relationship between GRACE score and serum VEGF before. Our study revealed that patients with high risk under GRACE stratification presented lower serum VEGF levels which suggested that low serum VEGF could predict poor cardiovascular outcomes.

Our study verified that low VEGF could predict poor clinical prognosis in CAD patients. Kaplan-Meier curves were depicted, and we found that patients with decreased serum VEGF levels had a higher possibility of adverse events. Our results were in agreement with those of the previous literature [6, 23]. In our multivariate Cox regression analysis, low VEGF and high Gensini score were independent risk factors for predicting poor clinical outcomes.

Some limitations should be acknowledged in our study. Prognostic analysis of serum VEGF was not performed in the SAP or ACS subgroup due to the insufficient sample size of both subgroups. Furthermore, we just collected those individuals without percutaneous coronary intervention (PCI) and coronary artery bypass graft (CABG), and our conclusions could not be applicable for those subjects who received PCI or CABG before. Furthermore, diagnosis-use assay was unavailable in this study, which hindered our results from using in the clinical practice early.

## 5. Conclusion

Serum VEGF exhibits efficient diagnostic value for detection of ACS from SAP with a cutoff value of 648.75 pg/mL. Low serum VEGF indicates severe coronary artery lesions, and higher GRACE score suggests poor clinical outcomes.

## Figures and Tables

**Table 1:** Baseline characteristics of study population.

	Control (*n* = 48)	CAD (*n* = 248)	*P* value
Age (years)	61.15 ± 8.02	61.54 ± 8.36	0.705
Sex (*n*, male)	23 (47.9%)	105 (42.3%)	0.475
Hypertension (*n*, %)	0 (0%)	126 (73.3%)	NS
Diabetes mellitus (*n*, %)	0 (0%)	53 (30.8%)	NS
Smoking (*n*, %)	0 (0%)	89 (51.7%)	NS
HbA1c (%)	5.97 ± 0.68	6.35 ± 1.21	0.009^*∗∗*^
CK-MB (U/L)	9.2 (7.7, 11.4)	11.0 (9.0, 13.4)	0.003^*∗∗*^
TnI	0.003 (0.001, 0.008)	0.007 (0.003, 0.023)	<0.001^*∗∗*^
TC	4.73 (3.98, 5.38)	4.69 (4.09, 5.48)	0.860
TG	1.21 (1.00, 1.67)	1.51 (1.16, 2.04)	0.011^*∗*^
HDL-c	1.35 (1.13, 1.60)	1.17 (1.02, 1.35)	0.002^*∗∗*^
LDL-c (mg/dL)	3.05 ± 0.63	2.88 ± 0.68	0.086
Cr (mg/dL)	70.06 ± 5.75	71.95 ± 9.27	0.066
LVEF (%)	60.33 ± 3.61	59.14 ± 5.42	0.145
Gensini score	NS	38 (17, 74)	NS
PCI (*n*, %)	NS	100 (40.3%)	NS
VEGF (pg/mL)	160.93 (121.09, 303.26)	645.57 (319.48, 901.14)	<0.001^*∗∗*^

Date are expressed as mean ± SD (standard deviation), median (interquartile range), and absolute number (percentage). *P* value for comparison between the CAD and the control group. ^*∗∗*^*P* < 0.01. HbA1c: hemoglobin A1c; CK-MB: creatine kinase-MB; TnI: troponin I; TC: total cholesterol; TG: triglyceride; HDL-c: high-density lipoprotein cholesterol; LDL-c: low-density lipoprotein cholesterol; Cr = creatinine; LVEF: left ventricular ejection fraction; PCI: percutaneous coronary intervention; VEGF = vascular endothelial growth factor.

**Table 2:** The values of VEGF, CK-MB, and TnT for predicting ACS against SAP.

	AUC (95% CI)	Cutoff value	Sensitivity (%)	Specificity (%)	*P* value
VEGF (pg/ml)	0.667 (0.559–0.735)	648.75	68.5	60.1	<0.001^*∗∗*^
CK-MB (U/mL)	0.622 (0.547–0.696)	25.95	60.7	54.4	0.002^*∗∗*^
TnI (ng/mL)	0.814 (0.748–0.897)	0.011	73.0	83.5	<0.001^*∗∗*^

^*∗∗*^ *P* < 0.01. AUC: area under the curve; CI: confidence interval; VEGF: vascular endothelial growth factor; CK-MB: creatine kinase-MB; TnI: troponin T; ACS: acute coronary syndrome; SAP: stable angina pectoris.

**Table 3:** Multivariable Cox regression analysis for predicting cardiovascular adverse events.

Variables	Univariable			Multivariable		
	HR	95% CI	*P* value	HR	95% CI	*P* value
Male	0.899	0.495–1.633	0.727			
Age	0.983	0.949–1.018	0.339			
Hypertension	2.831	1.318–6.080	0.008^*∗∗*^	2.435	1.130–5.248	0.023^*∗*^
Diabetes mellitus	1.403	0.695–2.833	0.345			
Smoking	0.654	0.360–1.187	0.162			
LVEF	0.954	0.914–0.996	0.033^*∗*^	0.956	0.914–1.000	0.048^*∗*^
CK-MB	0.996	0.980–1.011	0.591			
Creatinine	1.010	0.979–1.042	0.537			
LDL-c	0.049	0.000–1387212.1	0.762			
PCI	0.619	0.345–1.110	0.107			
Gensini score	1.006	1.002–1.009	0.001^*∗∗*^	1.006	1.002–1.009	0.002^*∗∗*^
Low VEGF	0.522	0.284–0.962	0.037^*∗*^	0.531	0.286–0.985	0.048^*∗*^

Variables were brought into multivariable analysis (*P* < 0.1). HR: hazard ratio; CI: confidence interval. ^*∗*^*P* < 0.05; ^*∗∗*^*P* < 0.01. LVEF: left ventricular ejection fraction; CK-MB: creatine kinase-MB; LDL-c: low-density lipoprotein cholesterol; PCI: percutaneous coronary intervention; VEGF: vascular endothelial growth factor.

## Data Availability

The data used to support the findings of this study are available from the corresponding author upon request.
